# Printability Metrics and Strain Rate Sensitivity of Multirole PVDF in Extrusion-Based Additive Manufacturing

**DOI:** 10.3390/polym17223085

**Published:** 2025-11-20

**Authors:** Nectarios Vidakis, Nektarios K. Nasikas, Nikolaos Michailidis, Maria Spyridaki, Nikolaos Mountakis, Apostolos Argyros, Vassilis M. Papadakis, Amalia Moutsopoulou, Markos Petousis

**Affiliations:** 1 Department of Mechanical Engineering, Hellenic Mediterranean University, 71410 Heraklion, Greece; vidakis@hmu.gr (N.V.); mspyridaki@hmu.gr (M.S.); mountakis@hmu.gr (N.M.); amalia@hmu.gr (A.M.); 2 Division of Mathematics and Engineering Sciences, Department of Military Sciences, Hellenic Army Academy, 16673 Vari, Greece; nasikas@sse.gr; 3 Physical Metallurgy Laboratory, Mechanical Engineering Department, School of Engineering, Aristotle University of Thessaloniki, 54124 Thessaloniki, Greece; nmichail@auth.gr (N.M.); aargyros@auth.gr (A.A.); 4 Centre for Research & Development of Advanced Materials (CERDAM), Centre for Interdisciplinary Research and Innovation, Balkan Centre, Building B’, 10th km Thessaloniki-Thermi Road, 57001 Thessaloniki, Greece; 5 Department of Industrial Design and Production Engineering, University of West Attica, 12243 Athens, Greece; v.papadakis@uniwa.gr; 6 Institute of Electronic Structure and Laser of the Foundation for Research and Technology-Hellas (IESL-FORTH), Hellas, N. Plastira 100m, 70013 Heraklion, Greece

**Keywords:** polyvinylidene fluoride (PVDF), three-dimensional (3D) printing, strain rate, elongation speed, Thermal evaluation, rheological evaluation

## Abstract

Recently, significant attention has been paid to the use of multirole materials in additive manufacturing (AM). Polyvinylidene fluoride (PVDF) is an ideal candidate material that has been selected for examination because of its unique characteristics. This study establishes a correlation between the macroscopic mechanical behavior and microscopic structural mechanisms, enabling the utilization of the deformation rate in tailoring the mechanical response of printed PVDF components. This research focuses on testing AM PVDF samples under different strain rates (10–300 mm/min), aiming to report their behavior under loading conditions compatible with the stochastic nature of real-life applications. The thermal (thermogravimetric analysis and differential scanning calorimetry) and rheological (viscosity and melt flow rate) properties were investigated along with their morphological characteristics (scanning electron microscopy). The response under combined dynamic and thermal loading was investigated through dynamic mechanical analysis, and the structural characteristics were investigated using spectroscopic techniques (Raman and energy-dispersive spectroscopy). The properties examined were the ultimate and yield strengths, modulus of elasticity, and toughness. Sensitivity index data are also provided. For completeness, the flexural strength, Charpy impact strength, and Vickers hardness were also evaluated, suggesting that the AM PVDF samples exhibit a resilient nature even when subjected to extremes regarding their strain rate versus their overall mechanical characteristics. PVDF exhibited a strain-hardening response with an increase in its strength of up to ~25% (300 mm/min) and a stiffness of ~15% (100 mm/min) as the loading speed of testing increased.

## 1. Introduction

Additive manufacturing (AM) is a versatile manufacturing technique that can be applied in several different fields, depending on the desired part to be manufactured or the specific application for each part to be utilized. Arguably, AM has various advantages and limitations compared to other manufacturing methods [1,2,3] and is related to the materials that are subject to the manufacturing spectrum of AM. This spectrum can be divided into seven main AM families: material extrusion (MEX) [4,5], vat photopolymerization (VPP) [6,7], powder-bed fusion (PBF) [8,9], material jetting (MJ) [10,11], binder jetting (BJ) [12,13], sheet lamination (SL) [14,15], and directed energy deposition (DED) [16,17].

All AM techniques are based on the deposition of successive material layers, thereby creating the desired three-dimensional part, with material extrusion (MEX) being the most commonly used [18]. Applications where AM has profound merits [19] are those related to medical [20,21,22] and dental [23] fields, aerospace [24] and automobiles [25], defense [26,27], food industry [28], electronics [29], and the oil and gas industry [30,31]. Each makes good use of the most suitable materials, which can take advantage of their unique properties and services.

Several options exist for material selection among the various AM techniques described above. This is because of the unique properties that each possesses and the relevant applications that they are going to be used in. Hence, a wide spectrum of materials used in AM can be categorized as metals [32,33], ceramics [34,35,36], polymers [37,38,39], their blends [40,41], or composites [42,43]. However, polymers have attracted much attention, mainly because of their favorable properties, ease of manufacture, and numerous capabilities and applications. Although many polymers can be employed, the most popular polymers used in AM are polylactic acid (PLA) [44,45,46], polypropylene (PP) [47,48,49], ABS [50,51,52], polyethylene terephthalate glycol (PETG) [53], poly(methyl methacrylate) (PMMA) [54], polyamide 12 (PA12) [55,56,57], and PC [58,59].

In everyday life, polymers are considered low-performing materials and are usually utilized in applications that do not require enhanced mechanical or physicochemical properties [60]. However, twin-screw reactive extrusion and MEX workflow have demonstrated that low-level copolymer addition can enhance the modulus, toughness, and impact resistance of printed polymer blends [61].

Recently, high-performance polymers have attracted considerable interest in the industrial field and have become a focal point for various research activities [62]. They are extensively used in many fields, such as medicine [63,64], dentistry [65], and aerospace [66]. Polysulfone (PSU), polyphenylsulfone (PPSU) [67], polyetheretherimide (PEI) [68,69], polyetheretherketone (PEEK) [70,71], polyphenylene sulfide (PPS) [72], polyimide (PI) [73], and polyvinylidene fluoride (PVDF) [74] are popular high-performance polymers used in AM applications.

Herein, we focus on PVDF, which is a widely used polymer in various applications. Regarding its market size, PVDF is yet to grow gradually in the future, as previously reported [75,76,77,78]. Based on the Market Research Future report, the PVDF market volume in 2023 was expected to be USD 49.22 billion and increase from USD 52.67 billion in 2024 to USD 90.49 billion in 2032, achieving 7% compound annual growth rate (CAGR) [79]. Techavio PVDF market analysis indicated that there would be a 6.3% CAGR within the forecast period of 2023–2028 [80].

Polyvinylidene fluoride (PVDF) belongs to the fluoropolymer family and is attractive because of its intriguing properties [81]. PVDF is generally considered a non-reactive thermoplastic polymer belonging to the fluoropolymer family, and is synthesized through the polymerization of vinylidene difluoride. This nonreactive nature of PVDF has been the subject of important applications in energy materials such as Li-ion battery separators and membranes [82,83]. PVDF is generally semi-crystalline and can be up to 50% amorphous in nature [84]. This has also provided the opportunity to utilize PVDF as a container for various solvents, liquid fuel tanks, and pipes for energy-source distribution [85]. The most common synthesis route for PVDF involves radical polymerization or copolymerization of vinylidene fluoride [86]. PVDF is known to possess a glass transition temperature of −40 °C and a molecular weight larger than 100.000 g/mol, while the melting and crystallization temperatures are 171–180 °C and 141–151 °C, respectively [87]. In addition, PVDF exhibits excellent resistance to weather conditions and exposure to the environment because it is UV-resistant [88] and exhibits antifouling properties [89].

PVDF exhibits excellent mechanical strength, thermal stability, processability, and chemical resistance [90], antioxidant properties, hydrolytic stability [90,91,92], and piezoelectric properties [87]. The most common applications of PVFDs are batteries [93], actuators [94], medical devices [95], ultrasonic transducers [96], and sensors [97,98], which can also be found in fields related to buildings and automobiles [99]. Owing to the diverse properties of PVDF (piezoelectric, electrical, chemical, and mechanical), it is considered a multirole high-performance polymer suitable for different types of applications or applications with diverse specifications [100,101].

PVDF is a widely used polymer in demanding applications owing to its robustness and performance in demanding environments. One such environment is the defense domain, where the need for high-performance, easy manufacturing, and recyclable parts is steadily growing [102,103]. PDVF has found some interesting applications in the defense domain, such as the 3D printing of hybrid rocket fuels [104], force transducers to account for behind armor blunt trauma (BABT) [105], metal-polymer composites for combustion and energy release [106,107,108,109], and sensors for monitoring the ballistic performance of concrete infrastructure [110]. A very promising property of PVDF, which can find numerous applications in the defense domain, is its piezoelectric property [111,112]. This property has enabled the fabrication of various sensors [113,114,115,116], and thus constitutes PVDF as a very important material for relevant applications.

Other uses of PVDF can be found in the biomedical domain, exhibiting an antimicrobial nature [117,118], in the food packaging industry [119], and in electronics, especially as nanogenerators, through the triboelectric phenomenon [120,121,122]. To date, very few studies have dealt in detail and in conjunction with each other with the most important physicochemical and mechanical characteristics of PVDF, aiming to shed more light on this very important material and, at the same time, look into the specificities arising from a novel manufacturing method, such as 3D printing.

In all of these real-life applications, the loads applied to the components have a stochastic nature [123]. Therefore, the response of materials under loads applied at different speeds is essential and provides valuable information for designing mechanism components. In this direction, research on the impact of strain rate on the response of materials and structures has been presented, providing valuable information for designing components in the defense domain, such as armor or propellants [124,125,126,127,128]. This is more critical in polymeric parts because of their viscoelastic behavior. Therefore, research on the response of high-performance polymers under various strain rates, such as poly(phthalazinone ether sulfone ketone) (PPESK) [129], with the aim of defense-related applications, is required. A fully 3D strain-rate–dependent Eyring-based model for PVDF that accurately captures both preyield viscoelasticity and postyield flow under various temperatures has been presented, providing a robust framework for interpreting our multi–strain-rate mechanical data [130]. Furthermore, an experimental demonstration of a 400% increase in PVDF’s d_33_ coefficient via controlled shear during extrusion offers a valuable comparison of processing-induced phase transformations and piezoelectric performance [131].

In AM, the 3D printed structure is an additional parameter that affects the response of the components. To address this issue, research has focused on examining the response of 3D printed standard engineering polymers, such as Polyamide 12 (PA12) [132], Polyethylene Terephthalate Glycol (PETG) [133], Polypropylene (PP), Polylactic Acid (PLA) [134], Thermoplastic Polyurethane (TPU), PMMA [135], and PC, under different strain rates [136]. In these studies, 3D printed parts were subjected to compressive or tensile uniaxial loads under different strain rates. The findings revealed different responses of the 3D printed polymeric parts when subjected to such types of loadings, and therefore justified the need for such investigations. Regarding 3D printed high-performance polymers, research is still limited to materials such as PSU and PPSU [67], as the bibliography research revealed.

The purpose of this research is to extensively study PVDF samples, focusing on their responses under different strain rates under uniaxial tensile loads. The research idea of reporting on MEX-produced PVDF parts tested under varying strain rates is novel because it combines several research areas (additive manufacturing, polymer mechanics, and dynamic loading performance). PVDF is well understood as a semicrystalline fluoropolymer; however, the processing of PVDF by MEX and understanding its performance at various strain rates have not yet been pursued. This research is important because the MEX process leads to anisotropic behavior, interlayer porosity, and microstructural heterogeneities, which will have a substantial effect on the strain rate effects on 3D printed components. Testing the changes in the mechanical response under varying strain rates will provide information on the deformation mechanisms in the 3D printed microstructure (i.e., strain hardening and viscoelasticity), which are critical for industries involving dynamic loads or impact conditions. In addition, these studies can also identify how the printing parameters/methodology produced concepts that demonstrate the rate-dependent behaviors that effectively tailor PVDF components for sensing, structural monitoring, or energy harvesting applications. Overall, the novelty of this research is the generation of a mechanistic understanding of how MEX-printed PVDF parts perform in dynamic mechanical environments, while simultaneously building on the knowledge of the additive manufacturing community for functional polymers and the basic understanding of polymer behavior under high-strain-rate loading environments.

Furthermore, for completeness of the characterization process, the PVDF samples were examined for their thermal and rheological behaviors and standard mechanical testing, with the aim of elucidating their intriguing mechanical properties. The PVDF raw material was transformed into a filament, which was subsequently 3D printed into predefined structures. Information regarding the thermal performance and properties was obtained through differential scanning calorimetry (DSC) (for material phase-change temperature determination) and thermogravimetric analysis (TGA) for degradation and residual mass estimation. The rheological characteristics are presented as viscosity graphs and melt flow rate (MFR) bars. Dynamic mechanical analysis (DMA) was also performed, presenting curves of storage and loss modulus, as well as damping factor, thus additionally revealing the response of the PVDF MEX AM parts under combined dynamic and thermal loadings.

For strain rate testing, various elongation speeds (10, 25, 50, 75, 100, 150, 200, 250, and 300 mm/min) were examined. The mechanical properties under investigation were tensile-related: the ultimate and yield strengths, modulus of elasticity, and toughness. The sensitivity index m was calculated. Furthermore, flexural strength, modulus of elasticity (stiffness), toughness, impact strength (Charpy), and Vickers hardness were assessed. The morphological characteristics of the samples were elucidated using electron microscopy (SEM) by capturing images of the fractured and side surfaces at different magnifications.

The examination of MEX 3D printed PVDF components under varying strain rates holds significant value from both practical and scientific perspectives. Practically, this study facilitates the optimization of PVDF components for applications subjected to dynamic or impact loading, such as flexible sensors, wearable electronics, structural health monitoring, and piezoelectric actuators. Given PVDF’s electroactive and piezoelectric properties, a comprehensive understanding of its mechanical response to strain rates, as presented in this study, is crucial to ensure durability, reliability, and consistent performance in practical applications. From a scientific standpoint, the findings of this research offer insights into the strain rate sensitivity and deformation mechanisms of semi-crystalline high-performance and additively manufactured multirole polymers, considering process-induced anisotropy and microstructural features introduced by MEX. Furthermore, this research addresses a critical gap in the mechanical characterization of functional polymers produced through extrusion-based additive manufacturing methods, where interlayer bonding, crystallinity, and filament orientation significantly influence mechanical performance. Thus, the significance of this work is twofold: it contributes to the design of functional 3D printed materials and enhances the understanding of the mechanical behavior of polymers under dynamic loading. The reported results can be exploited as a roadmap when designing PVDF parts to be manufactured using the MEX AM method.

## 2. Materials and Methods

**Figure 1:**
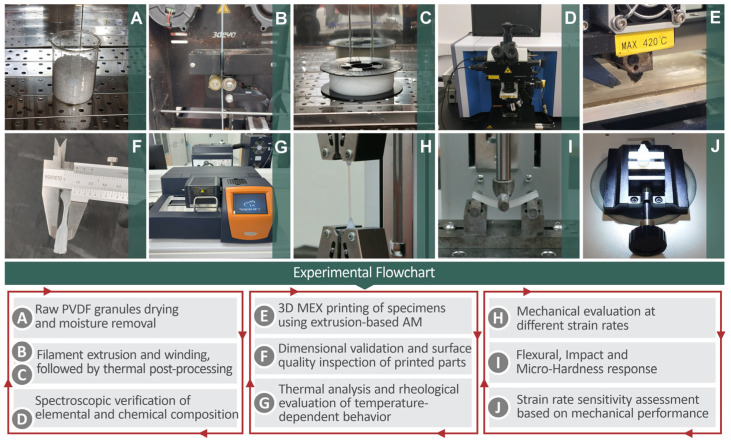
Visual representation of the experimental processes carried out in this research work, starting from the raw material to the testing of the 3D-manufactured specimens. The pictures presented in the top section correspond to the experimental flowchart in the bottom section.

The top section of Figure 1 shows images from the different phases of this research regarding material preparation and processing, filament, and subsequent 3D printed specimens. The thermal, rheological, mechanical, and morphological evaluations of each image are shown in the bottom section of the figure in an experimental flowchart. The entire procedure was as follows: raw PVDF in the form of granules underwent a drying procedure to remove any existing moisture (Figure 1A). Subsequently, PVDF filament extrusion was performed, followed by winding, thermal post-processing (Figure 1B,C), and elemental and chemical composition verification through spectroscopic methods (Figure 1D). The next step involved 3D printing of various PVDF specimens, which was performed through extrusion-based AM (Figure 1E), along with their dimensional validation and inspection of surface quality (Figure 1F), as well as thermal and rheological evaluation (Figure 1G). Finally, their mechanical evaluation was performed for various strain rates (Figure 1H), and their flexural, impact, and Vickers hardness responses (Figure 1I) were evaluated to assess the strain rate sensitivity and overall mechanical behavior.

### 2.1. PVDF Material Characteristics

PVDF was obtained from Jiangsu FreChem Co., Ltd. (Nanjing, China). PVDF in the form of granules, at this grade, according to the information provided in the material’s datasheet, has a density of 1.75–1.77 g/cm^3^, MFR of 6–12 g/10 min, tensile strength > 30 MPa (ASTM D638 [137]), elongation of 50–250% (ASTM D638), hardness (Shore D) of 70–80, and melting point of 160–174 °C.

### 2.2. PVDF Filament Extrusion and Pre-Printing Preparation

PVDF filament fabrication was performed using a 3devo Precision 450 extruder (Shanghai, China), considering the required settings and specifications corresponding to the PVDF characteristics. These characteristics were provided by the manufacturer, as well as the existing test results during the entire process. The temperatures of the four zones, starting from the hopper and reaching the nozzle, were 180 °C, 200 °C, 215 °C, and 220 °C, while the screw was set at 6 rpm, and the cooling fan was set to 50% capacity. The resulting filament was found to be 1.75 mm ± 0.05 mm in diameter, which was suitable for 3D printing. After extrusion, the filaments were prepared for the next step (feeding them into the 3D printer) by placing them in a conventional oven at 80 °C for 4 h, as part of a standard procedure, to dry.

### 2.3. PVDF Characterization: Raman, Rheology, Morphological, and Thermal

Multiple techniques were used to characterize PVDF, and the detailed methods are provided in the Supplementary File.

- Raman spectra were obtained using a spectrometer from HORIBA Scientific in Kyoto, Japan (LabRAM HR Raman spectrometer).
- Rheological characteristics, including viscosity and MFR, were assessed using a DHR-20 rotational rheometer (TA Instruments, New Castle, DE, USA). The MFR experimental procedure was implemented in accordance with the ASTM D1238 standard [138].
- Elemental analysis was performed using EDS (JSM-IT700HR-field-emission, Jeol Ltd., Tokyo, Japan), and SEM images were captured with a JSM 6362LV (Jeol Ltd., Peabody, MA, USA) in high-vacuum mode at 20 kV after Au sputtering of the PVDF 3D printed samples for morphological analysis.
- Thermogravimetric analysis (TGA) was conducted on a thermal analyzer by the company TA Instruments, located in New Castle, DE, USA (model named SDT 650 Discovery Simultaneous Thermal Analyzer).
- DSC was performed using a model named Discovery-Series DSC 25 by the TA Instruments company, established in Delaware, USA.

Thermal property measurements were carried out mainly to ensure that the processing temperatures in the study (extrusion, etc.) did not cause any thermal degradation of the materials, which would have affected the performance. The parameters used in the tests are listed in the Supplementary File.

### 2.4. 3D-Printed Specimens Manufacturing

The fabrication of 3D printed PVDF specimens was performed using a CreatBot F430 3D printer (Zhengzhou, China), with the following settings: nozzle temperature: 260 °C, build plate temperature: 150 °C, chamber temperature: ambient (~25 °C, unheated), nozzle diameter: 0.4 mm, layer height: 0.2 mm, printing speed: 25 mm/s, infill density: 100%, infill pattern: rectilinear (±45°), perimeter (outer walls): 2, part cooling fan: 0% (disabled). The specimens produced by 3D printing were analyzed for their tensile, flexural, and impact properties and were manufactured based on the relevant ASTM protocols and standards.

A rectilinear (±45°) infill was selected because it reduces anisotropy, as reported in the literature [139,140,141]. An appropriate raster orientation (e.g., alternating or cross-hatch patterns, such as ±45°) considerably reduces the anisotropy of the tensile response. Anisotropy is a common issue in 3D printed parts, affecting their mechanical behavior [142,143,144], regardless of the print orientation or material [145,146,147,148]. In particular, alternating raster directions allow for the applied tensile load to be shared more evenly by multiple filament orientations, encourage greater filament-to-filament bonding, and reduce the impact of any single raster direction in the load path. On the other hand, selecting 0° or 90° raster in tensile tests has been reported to optimize tensile strength when raster angles are aligned with the load direction [139,149]. Conducting tests on samples made at 0° and 90° for all the tested speeds would require an experimental effort beyond the limits of a single publication, as the samples would be tripled. Furthermore, the 3D printing settings were kept constant in the samples for comparison purposes. The effect on the parts’ response would require different values to be applied and the respective tests to be performed. However, this was not within the scope of this study.

### 2.5. Mechanical Properties Testing and Evaluation

This study examines the effect of strain rate on high-performance PVDF thermoplastics and assesses their mechanical response. The tests included uniaxial tensile (ASTM D638), impact (Charpy, ASTM D6110 [150]), flexural (ASTM D790 [151]), and microhardness (ASTM E384 [152]) tests. Dynamic Mechanical Analysis (DMA) further assessed the material under combined dynamic and thermal loading; details regarding the methodology of each experimental procedure are provided in the Supplementary File. Five repetitions were conducted for all the experiments. Furthermore, in the experimental results, error bars are included only in graphs that are linear and not in those showing exponential behavior.

Tensile tests on 3D printed PVDF specimens were conducted using an Imada MX2 (Toyohashi, Aichi, Japan) with standardized grips at speeds of 10 (standard value), 25, 50, 75, 100, 150, 200, 250, and 300 mm/min (room temperature) to represent a broad range of strain rates. These elongation speeds were selected to be in the range assessing the response of the PVDF 3D printed samples, from standard (10 mm/min) and low values up to quite high values, aiming to confront the stochastic nature of real-life loadings. The formulas considered for the calculation of the research metrics are presented in the Supplementary Materials. The elongation speeds and the corresponding strain rates are listed in Table 1.

## 3. Results

### 3.1. Raman and EDS Spectroscopy

**Figure 2:**
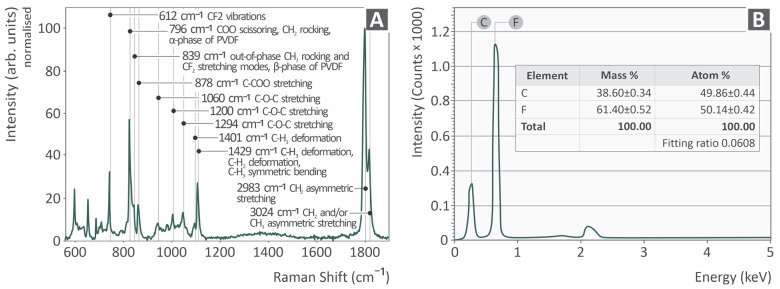
(**A**) Raman spectra of PVDF, (**B**) EDS results, and an inserted table presenting the elements found in the sample in mass % and atom %, respectively.

Figure 2A shows the Raman spectral signature of pure PVDF. In Table 2, the Raman peaks from the PVDF coupons are derived from the available literature and accompanied by the respective references. As shown in Figure 2B, the EDS results of the sample are presented, along with an inserted table containing the detected elements with their mass (%) and atom (%). As expected, fluorine is also detected.

The Raman spectrum of pure PVDF (Figure 2A) reveals several characteristic peaks corresponding to the specific vibrational modes of the polymer chains. The observed peaks and their assignments (Table 2) aligned closely with those of previous studies, confirming the structure and high quality of the material.

The intense peak at 612 cm^−1^ is attributed to the CF_2_ group vibrations of the PVDF backbone. The strong peak at 796 cm^−1^ corresponds to the COO scissoring and CH_2_ rocking modes, which are characteristic of the α-phase of PVDF. The medium-intensity peak at 839 cm^−1^ corresponds to the out-of-phase CH_2_ rocking and CF_2_ stretching modes, which are characteristic of the β-phase of PVDF, or to the result of post-synthesis processing, such as melt extrusion. In the present work, the β-phase content observed in commercial-grade PVDF is attributed primarily to processing via extrusion and not to the inherent crystal structure of the commercial-grade PVDF. The C-O-C and C-COO stretching modes at (878, 1060, 1200, and 1294 cm^−1^) reflect the presence of ether and carboxylate groups, possibly due to minor impurities or structural patterns in the polymer matrix. The CH_3_ and CH_2_ deformations are consistent with the aliphatic chain structure of the polymer. The very strong peak at 2983 cm ^−1^ is characteristic of CH_2_ asymmetric stretching vibrations, which are commonly observed in PVDF polymers. Finally, CH_2_ and/or CH_3_ asymmetric stretching at 3024 cm^−1^ further confirmed the presence of aliphatic groups in the polymer backbone vibration.

The presence of the β-phase and uniform elemental composition, as measured by Raman spectroscopy and EDS, suggests that the processed PVDF is structurally predisposed to exhibit improved tensile and toughness properties. This suggestion is supported by the results of the mechanical testing.

### 3.2. Thermal Properties of the PVDF Sample

**Figure 3:**
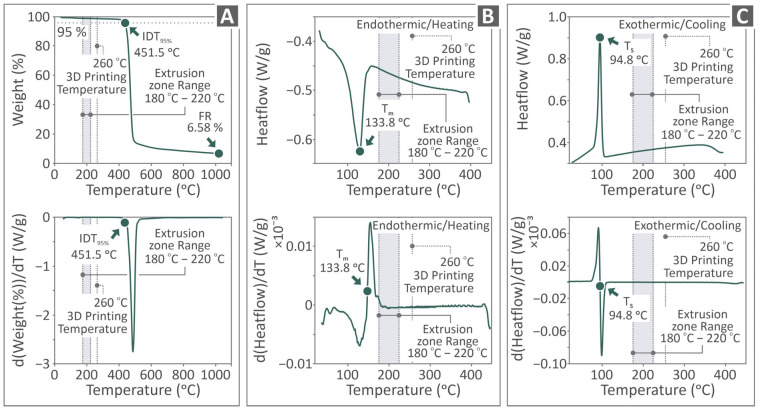
PVDF thermal properties: (**A**) TGA, and DSC (**B**) endothermic/heating testing, (**C**) exothermic/cooling testing, regular curves in the top row and their derivative in the row below.

Figure 3 shows the TGA and DSC curves originating from the thermal examination of the PVDF sample, with regular curves in the top row and their derivatives in the bottom row. The TGA-related curves are presented in Figure 3A as weight vs. temperature graphs, and the initial decomposition temperature (IDT) at 95%, 3D printing temperature, extrusion zone range, and final residue (FR) are shown in the same figure. Figure 3B shows the DSC curves for the endothermic/heating examination, along with the 3D printing temperature T_m_ and extrusion zone range. The corresponding results of the exothermic and cooling examinations are presented in Figure 3C, along with the 3D printing temperature, T_S,_ and extrusion zone range. In the figure, the temperatures employed for extrusion and 3D printing of the samples are indicated in the curves. As shown, the temperatures are higher than the melting point, whereas they are significantly lower than the temperature at which the material starts to thermally degrade. In Figure 3, in the TGA results, the T_onset_ (IDT_95%_) temperature was found to be 451.5 °C. The final residue (FR) was 6.58% (100% − 6.58% = 93.42% weight loss). In the DSC measurements, T_melting_ was found to be 133.8 °C while T_solid_ was found to be 94.8 °C.

### 3.3. Viscosity and Stress Curves vs. Shear Rate and MFR

**Figure 4:**
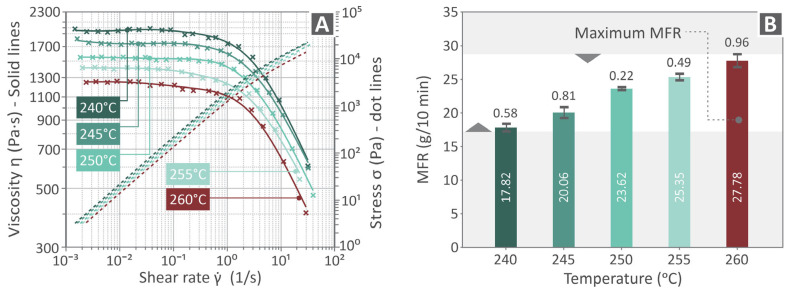
(**A**) Viscosity vs. shear rate and stress vs. shear rate presented by different colored-curves based on the corresponding testing temperature, (**B**) MFR bars per testing temperature.

To present the rheological characteristics of the PVDF samples, viscosity versus shear rate and stress versus shear rate curves, as well as MFR bars, are presented in Figure 4A and B, respectively. Each viscosity and stress curve, as well as the MFR bar, is represented by a different color depending on the temperature. It appears that as the viscosity decreases, the stress increases, whereas the MFR levels increase as the temperature increases.

### 3.4. DMA Results and Mechanical Properties of the PVDF Tested Samples

**Figure 5:**
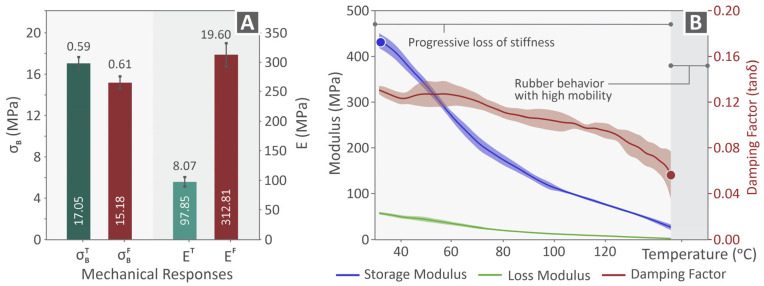
(**A**) Strength and modulus of elasticity levels after tensile and flexural testing of the PVDF sample tested under 10 mm/min elongation speed (standard value), (**B**) DMA data, storage modulus, loss modulus, and damping factor depicted in blue, green, and red curves, respectively (along with their deviation presented through the shaded zones around the curves).

Figure 5A shows some of the mechanical responses for which the PVDF samples were tested, considering those occurring at an elongation speed of 10 mm/min. The tensile and flexural properties are included, namely, the strength and modulus of elasticity, and the results indicate a very low tensile modulus in relation to the flexural modulus, whereas the strength properties are relatively close. The reference mechanical properties as tested and acquired herein are tensile strength: 17.05 ± 0.59 MPa, tensile modulus of elasticity: 97.85 ± 8.07 MPa, tensile toughness: 14.49 ± 0.52 MJ/m^3^, flexural strength: 15.18 ± 0.61 MPa, flexural modulus of elasticity: 312.81 ± 19.60 MPa, flexural toughness: 0.39 ± 0.02 MJ/m^3^, impact strength (Charpy): 13.34 ± 1.59 kJ/m^2^, Vickers hardness: 7.04 ± 2.04 HV. The DMA-derived information about the PVDF samples is provided in Figure 5B, including the curves of the storage modulus in blue, loss modulus in green, and damping factor in red. The bold and narrow curves represent the average values, whereas the color shading around the curves depicts the standard deviation of the acquired values. Moreover, the progressive loss of stiffness and rubber behavior in the high-mobility areas is indicated in the same DMA figure.

### 3.5. Tensile Testing Under Different Strain Rates

**Figure 6:**
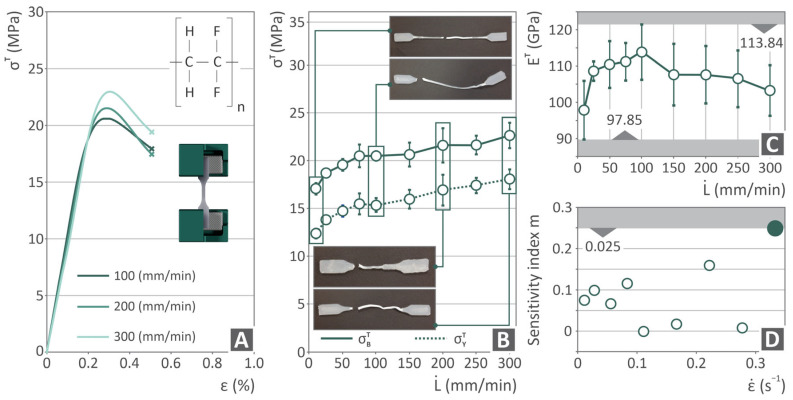
PVDF tensile testing results in the form of (**A**) tensile stress vs. strain curves for 100, 200, and 300 mm/min elongation speeds, accompanied by the PVDF chemical formula and a tensile testing illustration; (**B**) ultimate and yield strength vs. elongation speed curves in solid and dotted lines, respectively, and images of tensile tested samples tested with 10, 100, 200, and 300 mm/min; (**C**) tensile modulus of elasticity vs. elongation speed; and (**D**) sensitivity index m vs. strain rate.

In Figure 6, five different graphs show the information collected after tensile testing at different elongation speeds on the PVDF samples. Figure 6A shows the tensile stress–strain curves after tensile testing at elongation speeds of 100, 200, and 300 mm/min. In addition, there is an illustration of the testing and chemical formula of PVDF, where each curve is depicted in an altered color depending on the respective elongation speed.

Figure 6B illustrates the yield and ultimate strengths shown in the solid and dotted lines, respectively, versus the elongation speed, while four images are attached showing the various samples at elongation testing speeds of 10, 100, 200, and 300 mm/min. The two strength curves reveal an increase from 10 to 75 mm/min, then a decline up to 100 mm/min, and finally an increase again. The captured images indicate the ductile behavior of the samples, as all of them appeared to be significantly stretched. Figure 6C depicts the Young’s modulus vs. elongation speed data, revealing an increase from 10 to 100 mm/min, followed by a reduction. Figure 6D shows the sensitivity index m vs. the strain rate results, highlighting that for a 0.028 s^−1^ strain rate, the highest m value was achieved at a strain rate of 0.028 s^−1^.

**Figure 7:**
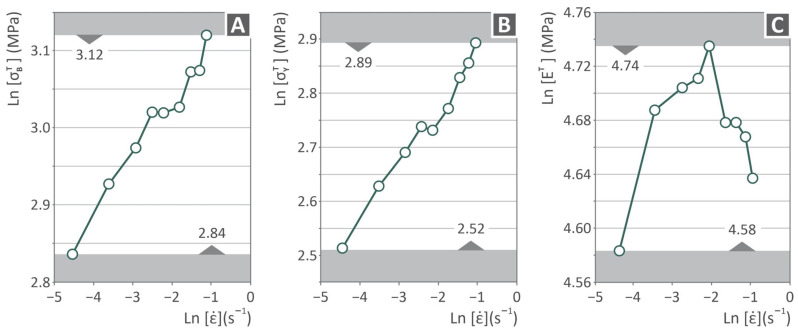
PVDF tensile testing-curves in ln scale: (**A**) ultimate, (**B**) yield strength, and (**C**) Young’s modulus, all vs. the strain rate.

Figure 7 presents a series of graphs containing PVDF tensile-related information, namely, ultimate strength (Figure 7A), yield strength (Figure 7B), and the tensile modulus of elasticity (Figure 7C) in MPa vs. the tensile strain rate in s^−1^, all on a ln scale. Both the ultimate and yield strengths continuously increase in all stages, while the modulus increases from −4.5 to −2.0 strain rate and then decreases from −2.0 to −1.0 strain rate. Ultimate strength begins at 2.84 MPa and reaches 3.12 MPa, yield strength begins at 2.52 MPa and reaches 2.89 MPa, and modulus begins at 4.58 MPa and reaches 4.74 MPa.

**Figure 8:**
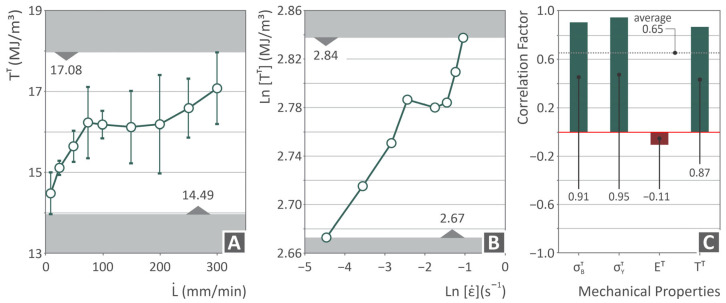
(**A**) tensile toughness vs. elongation speed, (**B**) ln(tensile toughness) vs. ln(strain rate), (**C**) correlation factor vs. mechanical properties graph.

Figure 8 shows more PVDF-related information in the tensile toughness versus elongation speed (Figure 8A) and ln(tensile toughness) versus ln(strain rate) (Figure 8B) graphs, both of which indicate a constant increase with an increase in the strain rate applied to the samples during testing. In addition, a graph of the correlation factor vs. the mechanical properties (Figure 8C) of the ultimate strength, yield strength, tensile modulus, and tensile toughness is shown. This metric (correlation factor) was computed using Pearson theory [119]. A positive value indicates that both parameters increase together, whereas a negative value indicates an inverse relationship. Only Young’s modulus showed negative values; for the other metrics, higher strain rates led to higher values, verifying the trend presented in the former graphs.

### 3.6. Morphology of the PVDF 3D Printed Samples Through SEM

**Figure 9:**
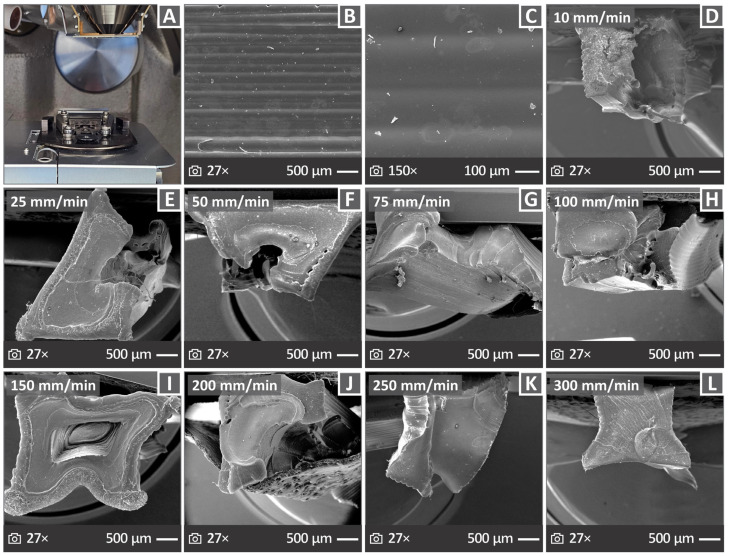
SEM images: (**A**) picture of the used apparatus, (**B**,**C**) Presentation of the samples’ side surface in 27× *g* and 150× *g* magnification, (**D**–**L**) presentation of the samples’ fractured surface in 27× *g* magnification, for those tested under 10, 25, 50, 75, 100, 150, 200, 250, and 300 mm/min elongation speeds, respectively.

**Figure 10:**
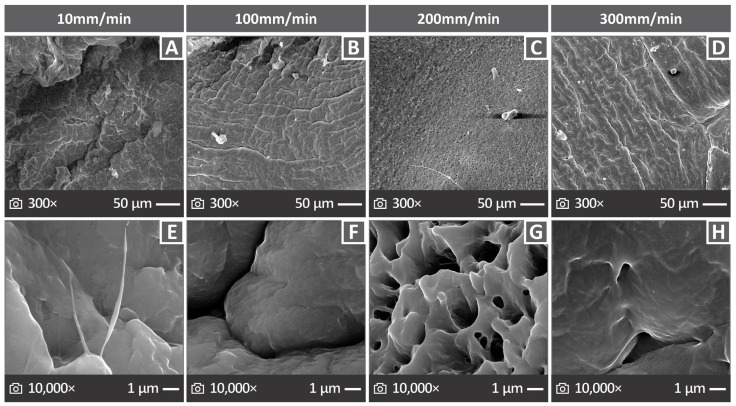
PVDF samples fracture surface images for those tested with 10, 100, 200, and 300 mm/min elongation speeds, in (**A**–**D**) 300× *g* and (**E**–**H**) 10,000× *g* magnifications.

Figure 9 presents the information collected through the SEM analysis of the 3D printed PVDF samples. Figure 9A shows the chamber of the SEM apparatus used for the analysis. Figure 9B,C show images collected from the side surface of the PVDF sample at 27× *g* and 150× *g* magnifications, respectively, indicating excellent layering quality and material deposition. Figure 9D–L show fractured surface images at 27× *g* magnification for the PVDF sample tested at all elongation speeds examined in this study. The samples exhibited ductile behavior, as they appeared to have been stretched before their fracture point, and a significant neck was formed. Figure 10 shows the SEM images of 3D printed PVDF samples fractured surfaces tested at 10 mm/min (Figure 10A,E), 100 mm/min (Figure 10B,F), 200 mm/min (Figure 10C,G), and 300 mm/min (Figure 10D,H) elongation speeds at magnifications of 300× *g* (top row) and 10,000× *g* (bottom row).

## 4. Discussion

PVDF is usually processed using conventional polymer processing techniques, whereas the application of AM techniques and their influence on physicochemical and mechanical properties remain less explored. PVDF, as mentioned before, exhibits a molecular weight that is approximately or even exceeds 100.000 g/mol [160]. Its molecular weight significantly contributes to its overall enhanced mechanical performance and stability [161]. This is also linked to the high electronegativity of fluorine atoms attached to the polymeric chain, as well as the high degree of bond dissociation energy with respect to the carbon-fluorine bonds [161]. This seems to affect the overall behavior of PVDF at various strain rates and shows ductile behavior even at the highest strain rate before breaking. This behavior is extremely important for applications that require the polymer to exhibit ductile behavior before failure, even at high strain rates [162]. This can be the case for applications where a specific part is subjected to multiple strain cycles, such as pipes or vibration-damping parts.

In liquid-crystalline materials, the percent crystallinity evaluated by DSC impacts the mechanical performance, including toughness, ductility, Young’s modulus, and yield strength. For example, making composites or blends with PVDF that have higher DSC-derived crystallinity will improve the mechanical properties owing to increased chain packing and decreased mobility of amorphous regions [163]. While greater crystallinity can improve the modulus and yield strength, it can also cause excessive brittleness and decreased overall toughness, but it may still have a high modulus [164]. The rigid crystal regions restrict the chain segment motion, and the minimal free volume improves the load transfer and yield strength, whereas the amorphous regions are responsible for the plastic deformation and yield point.

Because strain rate is treated as a continuous variable designed to test rate-dependent trends, applying discrete group-based hypothesis tests such as ANOVA would be statistically inappropriate. Instead, rate dependency was quantified using Pearson correlation analysis. This model confirms the viscoelastic response of the material by revealing strong positive relationships between the strain rate and properties such as the ultimate tensile strength, yield strength, and toughness. For example, the analysis highlights that the elastic modulus showed only a negligible negative correlation (r = −0.11), suggesting that the stiffness remained largely unaffected by the tested loading speeds. These quantitative findings, presented in Figure 5C, rigorously establish the significance of the observed rate-dependent behavior.

Figure 6 shows a gradual increase in the tensile strength with increasing strain rate. The yield strength exhibited a similar pattern. This can be attributed to strain-hardening effects on the PVDF polymer. Strain hardening refers to an increase in the stress required to continue plastic deformation of a material. In polymers, this behavior becomes more pronounced with increasing strain rate owing to both molecular and physical effects. When the strain rate increased, the polymer chains had less time to relax or reorient in response to the applied stress. As a result, there is greater alignment and stretching of the polymer chains along the direction of deformation. This alignment leads to an increase in entropic resistance to deformation, contributing to higher stress levels. Moreover, at high strain rates, localized deformation mechanisms, such as shear yielding, are suppressed, and instead, homogeneous plastic flow or strain-induced crystallization can dominate, depending on the polymer type. For semicrystalline polymers (such as polyethylene or PVDF), the strain rate may also promote crystallite orientation and even strain-induced crystallization, further reinforcing the material. In general, the polymer exhibits a transition from viscous-dominated to elastic-dominated behavior [165]. This phenomenon is crucial for applications involving impact or high-speed processing, in which mechanical stability under dynamic loading is essential.

In MEX 3D printed PVDF, the large error bars observed in the mechanical tests were mainly due to sample heterogeneity. Layer-by-layer deposition creates differences in the local crystallinity, porosity, and molecular orientation, which lead to variations in the mechanical behavior between nominally identical samples. Differences in raster alignment or small variations in interlayer adhesion create a significant difference in stress transfer and yield and thus cause a large standard deviation in the measured modulus and strength. This directly points to intrinsic anisotropy and the variation in the microstructure of 3D printed polymers as the primary source of experimental scatter. Other possible factors that could have contributed to the large deviation between samples in specific batches could have been measurement uncertainty or limited replicates. Regarding the measurement deviation, the devices used were certified to conform to the respective standards, and their accuracy was verified from time to time. Furthermore, the devices utilized in this study do not require calibration for each use, as their manufacturer states. Calibration was performed according to the manufacturer’s instructions regarding the time intervals to ensure the reliability and accuracy of the measurements. Regarding the number of replicates, instructions for the respective standards were followed in each test to ensure the reliability, validity, and reproducibility of the tests. As a result, these two factors, that is, measurement uncertainty and limited replicates, should be excluded because they cause large deviations between the samples in the mechanical tests. The only valid cause could be sample heterogeneity, which is a known issue in 3D printed samples, as reported in the literature [166,167].

In MEX 3D printed PVDF components, the strain-hardening behavior exhibited by the polymer at higher strain rates can be attributed to restrictions on the molecular mobility and the related microstructure evolution during deformation. Increases in strain rates limit the time required for the relaxation of the polymer chain segments. In this case, the density of the chain entanglements increases, and the molecular mobility decreases. The presence of entanglements facilitated the transfer of stress between adjacent chains, contributing to strain hardening. Likewise, at higher deformation rates, less free volume is available for segmental motion and molecular rearrangement, providing less opportunity for plastic flow and more energy storage in elastic deformation. Furthermore, crystallization can occur in PVDF. Alignment of chains at higher strain rates enables the formation of more ordered regions or extended-chain crystals, which also supports strain-hardening behavior and increased stiffness [168,169,170,171].

On the other hand, the Young’s modulus increased up to 100 mm/min (~15% increase compared to the value found at 10 mm/min, which is the standard value) and then started to decrease up to 300 mm/min, maintaining higher values than the one found at 10 mm/min (standard value). This finding was also verified in Figure 8C, in which, among all the mechanical properties tested for the 3D printed PVDF samples, only a negative correlation was observed for E^T^. The fact that the strength increases while the Young’s modulus decreases at high strain rates is illustrative of the interplay between the time-dependent viscoelasticity of the material and the type of structure formed from additive manufacturing. The Young’s modulus reflects the material’s initial stiffness under elastic deformation; this is largely dependent on the ability of the polymer to withstand small deformations. At higher strain rates, PVDF exhibited delayed relaxation behavior as a viscoelastic polymer. However, the very rapid loading at a higher strain rate does not allow the immediate elastic stiffness to be fully realized, owing to insufficient time for the intermolecular and chain segment movement to respond in cohesion in the early stages of deformation [172]. This resulted in an apparent lower modulus with a change in the ultimate strength.

The strain-rate sensitivity index (m) presented in Figure 6C quantitatively indicates the sensitivity of the material to the loading rate. The highest value was found at the highest strain rate tested, which also had the highest strength among the samples tested (increased by ~25% compared to the value found at 10 mm/min, which is the standard value, while the modulus of elasticity was ~5% higher). The layer-by-layer microstructure with variations in the chain orientation, crystallinity, and interlayer adhesion of the 3D printed samples also contributed to the strain rate sensitivity. A large m value in 3D printed PVDF suggests a significant influence of strain rate on the mechanical performance of the material, particularly under dynamic or impact loading conditions [172].

The behavior observed in Figure 7 and Figure 8 suggests that the PVDF samples manufactured through AM exhibit enhanced mechanical properties that show promise for applications in demanding environments, such as defense. The ability to utilize materials that can exhibit superior properties, complex geometries, and desired multifunctionalities for each specific application can open the path to novel applications and the translation of research results to actual capabilities. In this direction, a series of analogous multifunctionalities by producing composite materials based on high-performance materials is currently a very promising and intensive activity of this research group [173,174,175,176], with a special focus on defense [177]. Elucidation of the mechanical, rheological, thermal, and morphological characteristics of AM PVDF samples with respect to various stress/strain rates provides a complete and detailed picture of the various important characteristics that define the applications and overall behavior of PVDF. Having the ability to address these characteristics independently and in conjunction with each other, the research community can gain a better understanding of the various interdependencies occurring in AM PVDF samples, and thus be in a position to target the importance of each property to the overall behavior of AM PVDF samples. Studying additional aspects, such as the quantification of interfacial adhesion, can be the sole subject of future work, as it requires numerous tests and analyses [178,179].

The overall effect of the various strain rates on the 3D printed PVDF sample did not seem to impose any major hindrance on its mechanical properties, validating the superiority of using PVDF in demanding and extreme applications. This is also supported by the detailed SEM images, where a similar behavior was observed for the fracture surfaces of the various 3D printed PVDF samples subjected to the test. The fractured surfaces appeared to be free of cavitation or anomalous behavior. Ductile behavior was observed on the fractured surfaces, and all samples showed the same behavior. This is in agreement with previous studies [173,174,175,176] that reported an analogous behavior accompanied by extensive cavitation. This behavior was not observed in this work, probably because of the AM method, which deposits successive layers that are interconnected on large surfaces, and not a bulk piece of PVDF, which would be more prone to cavitation upon strain.

In general, 3D printing of PVDF samples is a reliable method for manufacturing various parts for applications in extreme environments and could possibly open up novel pathways for applications in the defense and aerospace industries. In this domain, toughness is a critical factor, and the findings herein suggest that toughness is enhanced by an accelerated strain rate. This is probably due to creep between the polymeric chains that contain fluoride branches, which tend to interlock and interact strongly. The alignment of the polymeric changes under external strain can induce orientation to the polymeric changes, thus acting as a reinforcement agent for the strained material. This behavior has been found to be very important for the preparation of super-tough PVDF-based multirole materials [180].

## 5. Conclusions

In this study, we attempted to provide new insights into the response of AM PVDF parts. Polyvinylidene fluoride (PVDF), a multirole polymer, is employed in various industrial fields. Therefore, knowing its response under different strain rates provides valuable information for real-life applications in which loadings have a stochastic nature. The raw PVDF material was extruded into filaments and then converted into 3D printed samples, which constituted the basis of this study. These samples were suitable for a series of experiments and tests focused on elucidating the various intriguing properties of the AM PVDF samples. Raman analysis and EDS were performed to provide data that would clarify and explain the chemical compositions of the examined samples. To analyze the thermal characteristics, thermogravimetric analysis (TGA) and differential scanning calorimetry (DSC) were used to broaden the knowledge about the sample reaction at high temperatures and separate the resulting phases. The rheological behavior of the AM PVDF samples was revealed through viscosity and MFR data, and DMA was performed for combined thermal and mechanical loading assessments. During the tensile tests, the ultimate and yield strengths, Young’s modulus, and toughness were assessed after subjecting the AM PVDF samples to nine elongation speeds. The flexural strength, modulus of elasticity, and toughness were also investigated using research-derived scientific data, together with the impact strength (Charpy) and Vickers hardness. SEM was used for the morphological inspection of the manufactured samples, providing potentially useful information about the effect of different strain rates on the structure and morphology of the specimens. Overall, an in-depth analysis is provided, yielding novel information about the interdependencies of the various mechanical, rheological, thermal, and physicochemical characteristics of the AM PVDF samples. The main findings are summarized as follows.

- The AM PVDF samples exhibited typical (for polymers) strain-hardening behavior at higher strain rates.
- The tensile strength and Young’s modulus increased by ~25% and 5%, respectively, at the highest elongation speed (300 mm/min).
- The ductility of the samples was not affected by the increase in the testing speed.
- The toughness was also increased with the increase in the testing speed.
- The highest Young’s modulus (stiffness) was found at an elongation speed of 100 mm/min, which was approximately 15% higher than the lowest value found at 10 mm/min.

The key finding of strain-rate effects research on MEX 3D printed PVDF parts is that high strain rates strongly improve strain hardening, partly due to microscopic structural mechanisms. The mechanical performance of printed PVDF may be tailored by considering both the strain rate and the microstructure obtained during printing for their effect on the macroscopic properties. This insight offers a pathway for the development of high-performance functional components, particularly for applications that require high resistance and durability.

Future work could include the strengthening of the multifunctionality of PVDF 3D printing through the formation of composites with special and focused properties, such as increased strength or antibacterial performance, owing to the qualities of the PVDF matrix material, which is already used in the medical field. Furthermore, additional high-performance polymers should be tested under a range of strain rates, as this information is crucial and should be taken into consideration when developing components to be built with these materials using the AM method. For completeness, further characterization methods, such as interfacial adhesion, FTIR/XRD, and oscillatory rheology, are required. Studies on the anisotropy of the samples or the effect of the 3D printing parameters of the samples on the mechanical performance can also be carried out.

## Figures and Tables

**Table 1:** List of the utilized speed of elongation and the respective strain rate.

Speed of Elongation (mm/min)	Strain Rate (s^−1^)	Speed of Elongation (mm/min)	Strain Rate (s^−1^)	Speed of Elongation (mm/min)	Strain Rate (s^−1^)
10	0.011	75	0.083	200	0.222
25	0.028	100	0.111	250	0.278
50	0.056	150	0.167	300	0.333

**Table 2:** PVDF pure Raman peaks (significant) and their respective assignments.

Wavenumber (cm^−1^)	Intensity	Raman Peak Assignment
612	Strong	CF2 vibrations [153]
796	Strong	COO scissoring [154]; CH_2_ rocking, α-phase of PVDF [153]
839	Medium	out-of-phase CH_2_ rocking and CF_2_ stretching modes, β-phase of PVDF [153]
878	Medium	C-COO stretching [155]
1060	Weak	C-O-C stretching [153]
1200	Medium	C-O-C stretching [156]
1294	Medium	C-O-C stretching [153]
1401	Weak	C-H_3_ deformation [157]
1429	Strong	C-H_3_ deformation [157,158]; C-H_2_ deformation [157,158]; C-H_3_ symmetric bending [155,156,157];
2983	Very Strong	CH_2_ asymmetric stretching [159]
3024	Strong	CH_2_ and/or CH_3_ asymmetric stretching [154]

## Data Availability

The raw/processed data required to reproduce these findings cannot be shared because of technical or time limitations.

## Supplementary Materials

The following supporting information can be downloaded at: https://www.mdpi.com/article/10.3390/polym17223085/s1, Theory and methodology followed. S1. Calculation formulas for the metrics considered herein; S2. Raman; S3. Rheology, EDS, and SEM; S4. TGA and DSC analyses; S5. Mechanical Testing.
