# Supplementary material for: Printability Metrics and Strain Rate Sensitivity of Multirole PVDF in Extrusion-Based Additive Manufacturing
Source: Polymers (Basel). 2025 Nov 20;17(22):3085. doi: 10.3390/polym17223085 (PMC12656586; doi:10.3390/polym17223085)
Supplement: Supplementary file 1 [file polymers-17-03085-s001.zip › polymers-3944365-supplementary.pdf]

polymers

MDPI

Supplementary for Article

Printability Metrics and Strain Rate Sensitivity of Multirole PVDF in Extrusion-Based Additive Manufacturing

Nectarios Vidakis [1], Nektarios K. Nasikas [2], Nikolaos Michailidis [3,4], Maria Spyridaki [1], Nikolaos Mountakis [1], Apostolos Argyros [3,4], Vassilis M. Papadakis [5,6], Amalia Moutsopoulou [1] and Markos Petousis [1,*]

[1] Department of Mechanical Engineering, Hellenic Mediterranean University, 71410 Heraklion, Greece; vidakis@hmu.gr (N.V.); mspyridaki@hmu.gr (M.S.); mountakis@hmu.gr (N.M.); amalia@hmu.gr (A.M.)
[2] Division of Mathematics and Engineering Sciences, Department of Military Sciences, Hellenic Army Academy, 16673 Vari, Greece; nasikas@sse.gr
[3] Physical Metallurgy Laboratory, Mechanical Engineering Department, School of Engineering, Aristotle University of Thessaloniki, 54124 Thessaloniki, Greece; nmichail@auth.gr (N.M.); aargyros@auth.gr (A.A.)
[4] Centre for Research & Development of Advanced Materials (CERDAM), Centre for Interdisciplinary Research and Innovation, Balkan Centre, Building B', 10th km Thessaloniki-Thermi road, 57001 Thessaloniki, Greece
[5] Department of Industrial Design and Production Engineering, University of West Attica, 12243 Athens, Greece; v.papadakis@uniwa.gr
[6] Institute of Electronic Structure and Laser of the Foundation for Research and Technology-Hellas (IESL-FORTH), Hellas, N. Plastira 100m, 70013 Heraklion, Greece
* Correspondence: markospetousis@hmu.gr; Tel.: +30-281-0379-2272

Abstract

Recently, significant attention has been paid to the use of multirole materials in Additive Manufacturing (AM). Polyvinylidene fluoride (PVDF) is an ideal candidate material that has been selected for examination because of its unique characteristics. This study establishes a correlation between the macroscopic mechanical behavior and microscopic structural mechanisms, enabling the utilization of the deformation rate in tailoring the mechanical response of printed PVDF components. This research focuses on testing AM PVDF samples under different strain rates (10-300 mm/min), aiming to report their behavior under loading conditions compatible with the stochastic nature of real-life applications. The thermal (thermogravimetric analysis and differential scanning calorimetry) and rheological (viscosity and melt flow rate) properties were investigated along with their morphological characteristics (scanning electron microscopy). The response under combined dynamic and thermal loading was investigated through dynamic mechanical analysis, and the structural characteristics were investigated using spectroscopic techniques (Raman and energy-dispersive spectroscopy). The properties examined were the ultimate and yield strengths, modulus of elasticity, and toughness. Sensitivity index data are also provided. For completeness, the flexural strength, Charpy impact strength, and Vickers hardness were also evaluated, suggesting that the AM PVDF samples exhibit a resilient nature even when subjected to extremes regarding their strain rate versus their overall mechanical characteristics. PVDF exhibited a strain-hardening response with an increase in its strength of up to ~25% (300 mm/min) and a stiffness of ~15% (100 mm/min) as the loading speed of testing increased.

Keywords: Polyvinylidene fluoride (PVDF); Three-dimensional (3D) printing; Strain rate; Elongation speed; Thermal evaluation; Rheological evaluation

S1. Calculation formulas for the metrics considered herein

The following formulas were considered for the calculation of the sizes utilized herein:

σ^T: $Tensile\ stress\ (MPa)$, calculated by: $\sigma^T = \frac{F}{A_0}\ (MPa)$ (1)

where: F: $Applied\ tensile\ force\ (N)$ and A_0: $Nominal\ cross-section\ area\ (mm^2)$

ε: Strain (mm/mm), calculated by: $\varepsilon = \frac{\Delta L}{L_0}(mm/mm)$ (2)

where: ΔL: $Specimen\ elongation\ (mm)$, and L_0: $Initial\ specimen\ length\ (mm)$ ($L_0 = 15\ (mm)$)

σ_B^T: $Tensile\ ultimate\ strength\ (MPa)$, is found by the formula: $\sigma_B^T = \max(\sigma^T)\ (MPa)$ (3)

$\dot{L}$: $Elongation\ speed\ (mm/min)$ is: $\dot{L} = \{10, 25, 50, 75, 100, 150, 200, 250, 300\}\ (mm/min)$

E^T: $Tensile\ modulus\ of\ elasticity\ (MPa)$

$\dot{\varepsilon}$: $Tensile\ strain\ rate\ (s^{-1})$, calculated by: $\dot{\varepsilon} = \frac{\dot{L}}{L_0 \cdot 60}(s^{-1})$ (4)

m: $Strain\ rate\ sensitivity\ index$, calculated by: $m = \frac{\ln(\sigma_{B,i}^T/\sigma_{B,i-1}^T)}{\ln(\dot{\varepsilon}_i/\dot{\varepsilon}_{i-1})}, i = 2, 3, \dots, n$ (5)

where:
$\sigma_{B,i}^T, \sigma_{B,i-1}^T$: $Tensile\ ultimate\ strength\ values\ for\ two\ successive\ strain\ rates\ (MPa)$
and
$\dot{\varepsilon}_i, \dot{\varepsilon}_{i-1}$: $Strain\ rates\ corresponding\ to\ the\ tensile\ ultimate\ strength\ values\ \sigma_{B,i}^T$ $and\ \sigma_{B,i-1}^T (s^{-1})$
And n: $Number\ of\ tested\ elongation\ speeds$ is $n = \#(\dot{L})$ (6)

σ_Y^T: $Tensile\ yield\ strength\ (MPa)$

T^T: $Tensile\ toughness\ (MJ/m^3)$ is: $T^T = \int_0^{\varepsilon_{max}} \sigma^T(\varepsilon) d\varepsilon\ (MJ/m^3)$ (7)

where: ε_{max}: $Maximum\ strain\ (mm/mm)$ is calculated by: $\varepsilon_{max} = \max(\varepsilon)\ (mm/mm)$ (8)

$\dot{\varepsilon}_{max(m)}$: $Strain\ rate\ at\ maximum\ strain\ rate\ sensitivity\ index\ m\ (s^{-1})$, is calculated by:

$\dot{\varepsilon}_{max(m)} = \dot{\varepsilon}_i\ where\ m_i = max\{m_1, m_2, \dots, m_n\}, i = 1, 2, \dots, n\ (s^{-1})$ (9)

where: m_{max}: $Maximum\ strain\ rate\ sensitivity\ index$
which is: $m_{max} = max\{m_1, m_2, \dots, m_n\}, i = 1, 2, \dots, n$ (10)

S2. Raman

A LabRAM HR Raman Spectrometer (HORIBA Scientific, Kyoto, Japan) with a 785 nm laser and 400 mW maximum output was used to acquire Raman spectra. The setup included a grating with 600 grooves/mm, a 400 μm confocal pinhole, and an Olympus (Tokyo, Japan) LMPlanFL N objective lens with 50× magnification at a 10.6 mm working distance. The laser power on the sample was limited to 90 mW using a Neutral Density filter. Measurement volume was 1.7 μm laterally and 2 μm axially. Raman spectra ranged from 40 to 1900 cm^{-1}, with each point exposed for 2 s and five accumulations. No sample damage was observed. Raw data were processed in LabSpec software (HORIBA Scientific, Kyoto, Japan): removing cosmic rays, denoising with a 5-point kernel, background

removal with a 5th-grade polynomial, normalizing by maximum peak, and cropping between 200 and 1900 cm^{-1}.

S3. Rheology, EDS, and SEM

A rotational rheometer, DHR-20 rotational rheometer (Discovery Hybrid Rotational Rheometer) (TA Instruments, New Castle, Delaware, United States) was used for rheological examination. It consisted of two parallel plates, as well as a temperature-controlled environmental test chamber. The rheometer featured a torque range of 1–100,000 μNm and used 25 mm diameter plates with a 1 mm gap. Temperature range was 240-260 ^{o}C, shear rate was 0.001 - 100 1/s, acquisition time was set at 10 s, and the measurements tolerance was 3%.

A melt flow index was also implemented based on ASTM D1238-13 for the MFR. EDS was performed using a field-emission SEM JSM-IT700HR (Jeol Ltd., Tokyo, Japan) machine. Morphological evaluation was performed using a JSM 6362LV by Jeol Ltd. (Peabody, Massachusetts, United States) apparatus (mode: high-vacuum, 20 kV, Au sputtering of PI 3D printed coupons).

S4. TGA and DSC analyses

TGA was performed utilizing a model named SDT 650 Discovery Simultaneous Thermal Analyzer by the company TA Instruments (New Castle, Delaware, United States). Indentation was carried out at a range of 25-1050°C. These results confirmed that the temperatures used for 3D printing did not exceed these levels and caused material degradation. Endothermic and exothermic DSC were both conducted with the assistance of a TA Instruments Discovery-Series DSC 25 apparatus (Delaware, USA). A nitrogen atmosphere was used in measurements. DSC parameters were scan rate: 15°C/min, temperature range: 30°C to 400°C, gas: N_2, gas flow: 50 ml/min.

S5. Mechanical Testing

The aim herein was to evaluate the effect of the speed a load is applied (strain rate) on the performance of the high-performance PI thermoplastic and report its response. Still, for completeness, mechanical tests we carried out according to respective standards, for uniaxial (tensile, ASTM D638), flexural (three-point-bending, ASTM D790), and impact (Charpy, notched, ASTM D6110) loadings, and microhardness (M-H, ASTM D384).

Uniaxial and flexural tests were carried out on an Imada MX2 apparatus, Aichi, Japan, with a 10 mm/min test speed and 52mm clearance in the flexural test. Charpy notched impact testing was conducted using a Terco MT220 (Terco, Kungens, Sweden), setting the hammer release height at 367 mm. M-H measurements were acquired with an Innova Europe Vickers 300 apparatus, adjusted at 100-gF load, on polished samples, with a 10-s indentation. Five samples were tested per case in ambient room conditions.

In addition to static loading tests, Dynamic Mechanical Analysis (DMA) was conducted to evaluate the response of the PI thermoplastic under combined dynamic mechanical (three-point-bending) and thermal loading. DMA was performed using a DHR20 Discovery Hybrid Rheometer with a 3-point bending geometry and a 40 mm span. The bending samples measured 10 mm in width, 50 mm in length, and 3 mm in height. The analysis covered a temperature range of 30–160°C with a heating rate of 5°C/min. During the test, sinusoidal strains ranging from 0 to 0.05% were applied at an oscillation frequency of 1 Hz. The force-tracking system maintained consistent contact with the sample throughout the test.
